# Association between neuropeptide Y gene polymorphism and antipsychotics effect

**DOI:** 10.3389/fpsyt.2022.1014952

**Published:** 2022-10-21

**Authors:** Qian Zhang, Yajie Wan, Xinzhe Du, Yao Gao, Xiao Wang, Kewen Wu, Xiaohu Zheng, Yu Wang, Cheng Zhao, Li Li, Xianju Guo, Xinrong Li, Sha Liu, Yong Xu

**Affiliations:** ^1^Department of Psychiatry, First Hospital of Shanxi Medical University, Taiyuan, China; ^2^Shanxi Key Laboratory of Artificial Intelligence Assisted Diagnosis and Treatment for Mental Disorder, First Hospital of Shanxi Medical University, Taiyuan, China; ^3^Shanxi Province Social Welfare Kangning Psychiatric Hospital, Jinzhong, China; ^4^Yangquan Mental Health Hospital, Yangquan, China; ^5^Changzhi Mental Health Center, Changzhi, China; ^6^Gaoping Disabled Persons' Federation Mental Rehabilitation Hospital, Gaoping, China; ^7^The Second Hospital of Yangquan Coal Industry Group Co. Ltd., Yangquan, China; ^8^Heping Hospital Affiliated to Changzhi Medical College, Changzhi, China; ^9^Department of Mental Health, Shanxi Medical University, Taiyuan, China

**Keywords:** schizophrenia, NPY, gene polymorphism, antipsychotics, drug response

## Abstract

**Objective:**

The pathogenesis of schizophrenia is associated with neuropeptide Y (NPY) gene polymorphism to explore the relationship between rs16141, rs16145, and rs5573 polymorphisms in the NPY gene and antipsychotics response in the Chinese population.

**Methods:**

The unrelated 228 Chinese Han patients with schizophrenia were enrolled in the present study. Genotypisation within NPY gene was performed using the KASP genotyping assays. Before treatment and on the weekends of the 2nd, 4th, and 8th weeks after treatment, the medication status of the patients was recorded and the positive and negative syndrome scale (PANSS) was used to evaluate the clinical effect. A reduction in total PANSS scores ≥50% were classified as good responders, while others were poor responders. We evaluated the association between NPY gene and antipsychotic efficacy by comparing allele and genotype distribution, correlation analysis, linkage imbalance, and five genetic models between the two groups.

**Results:**

No significant associations were found in the rs16141, rs16145, and rs5573 of NPY and antipsychotic treatment response (all *p* > 0.05). There was no significant relationship between the three SNPs polymorphisms in the NPY gene and the changes of positive, negative and general psychopathology subscales scores at each stage (all *p* > 0.05). The distribution of genotype and allele frequencies of locus rs16141 was not statistically difference between good responders and poor responders (genotype: χ2 =4.088, *p*=0.043, *p-correction* = 0.129; allele: χ^2^ = 4.088, *p* = 0.027, *p-correction* = 0.081). The allele distribution of rs5573 was significantly different between groups, yet the difference was disappeared after correcting (χ^2^ = 4.136, *p* = 0.042, *p-correction* =0.126). The distribution frequencies of TA/TG and GG haplotypes constituted by rs16141 and rs5573 showed no statistical difference between the two groups (*p* > 0.05). In recessive inheritance mode, NPYrs5573 was found to be associated with antipsychotic drug response (G/G vs. A/A +A/G: *p* = 0.028, AIC = 197.2, BIC = 210.9).

**Conclusions:**

This study didn't found association between polymorphisms in the NPY gene locus (rs16141, rs16145, and rs5573) and the response to antipsychotics after Bonferroni correction. The polymorphism of NPY gene and the efficacy of antipsychotic drugs in patients with schizophrenia need further study.

## Introduction

Schizophrenia is a serious and persistent neuropsychiatric disorder whose core symptoms are positive symptoms, negative symptoms and cognitive impairment (1). Schizophrenia has profound effects on individuals and societies, and the lifetime prevalence of the world population is about 1% (2). Schizophrenia usually appears in early adulthood, but studies have shown that its pathogenesis begins in early neurodevelopment (3). Many studies have shown that genetic and environmental factors (4) can lead to damage to neural tissue during development, which in turn contributes to the development of schizophrenia (5). Drug therapy is an effective treatment for schizophrenia, and most patients usually require long-term antipsychotic medication (6). However, different patients show significant differences in drug efficacy and adverse reactions (7). Only about one-third of patients show good efficacy, and drug side effects affect treatment adherence and prognosis (8). Studies have shown that gene polymorphisms play an important role in individual differences in drug efficacy, or can be used as important markers for drug efficacy prediction to guide clinical medication (9).

The neuropeptide Y (NPY) gene is a candidate gene for schizophrenia research. The human NPY gene is located in the chromosome 7 and encodes 36 amino acid residues (10). NPY is widespread in the central and peripheral nervous systems of humans and many animals. NPY has a wide range of functions and plays an important role in the food intake, sexual behavior, information processing, cognition, learning and memory, control of blood pressure, sympathetic excitability, regulation of stress and anxiety (11). There is growing evidence that NPY plays an important role in the pathophysiology of schizophrenia. Studies have found decreased NPY mRNA expression in the prefrontal cortex of the brain in patients with schizophrenia. A study found that the expression of NPY is dependent on the regulation of BDNF, which in turn is fine-tuned through a microRNA (miRNA)-mediated mechanism (12). A follow-up study showed that the level of NPY in the cerebrospinal fluid (CSF) of patients with schizophrenia was related to longitudinal outcome, and NPY levels was also related to the Social Competence of the patients (13). The NPY system regulates central dopamine signaling, which is closely related to the pathophysiology of psychotic symptoms (14). Activation of the NPY receptor Y2 subtype appears to play a key role in mediating such dopaminergic effect. An animal study has shown that mice treated with a Y2 receptor inducer can induce a variety of behavioral abnormalities. Signaling of the NPY receptor Y2 subtype modulates many behavioral and cognitive functions associated with schizophrenia and psychotic disorders (15). These studies suggest that NPY gene plays an important role in the development of schizophrenia.

Several studies have examined the relationship between antipsychotic drugs and NPY levels. For example, X Chen et al. (16) found an inverted U-shape dose-response effect for olanzapine doses ranging from 1 μM to 100μM, where 10 and 25 μM olanzapine increased NPY concentrations. An animal study found no change in NPY mRNA levels in rats treated with antipsychotic drugs, but increased NPY receptor type 1 (Y1R) mRNA levels, reflecting increased expression of this receptor protein, particularly in the lateral hypothalamus (17). In a study of the effects of clozapine serum concentrations on a variety of appetite-regulating peptides, an inverse association was found between NPY levels and clozapine dose (18). However, another study found no association between NPY levels and clozapine (19).

Currently, the relationship between antipsychotic efficacy and NPY gene polymorphisms is unclear. Therefore, this study selected the single nucleotide polymorphism sites of NPY gene that have been reported in previous studies related to schizophrenia, and explored their association with the efficacy of antipsychotic drugs in Chinese Han population.

## Materials and methods

### Subjects

This study comes from the “Schizophrenia molecular typing and individualized diagnosis and treatment research” project leaded by “Shanxi Schizophrenia Collaboration Group.” The project focuses on genetic research and pharmacogenomics research on schizophrenia markers. A total of 7 mental health hospitals in Shanxi Province participated. The participants are planned to enroll between January 2017 to December 2021. This is a multi-center, multi-phase study with the objectives of: genomics of antipsychotic efficacy, molecular markers of treatment-resistant schizophrenia (TRS), molecular markers of adverse effects of antipsychotics, and common schizophrenia.

The project has a principal leader, a research coordinator (responsible for coordination across hospitals), and multiple investigators from each hospital (responsible for recruitment, clinical assessment, blood sample collection, and data digitization). All researchers are professionally trained. Researchers will report on research progress, questions and recommendations monthly to ensure protocol implementation and research quality.

This research project includes three parts: (1) Genomic studies of antipsychotic efficacy: Screening subjects who meet the entry requirements, if a single antipsychotic drug is used, can be included in this section. The basic information of the subjects, medication status and blood samples were mainly collected, the Positive and Negative Syndrome Scale (PANSS) of the patients at baseline was assessed, and follow-up was conducted in 2nd, 4th, 8th week. (2) Molecular marker study of TRS: This part is included when the patient belongs to TRS. The detailed medication status was recorded and divided into retrospective studies and prospective studies according to whether new drugs were about to be used. The PANSS at baseline and 6th week was mainly recorded. (3) Molecular markers of adverse reactions of antipsychotic drugs: If they do not meet the inclusion criteria of any of the above subgroups, they will be included in the general schizophrenia group. The adverse reactions of patients after medication were recorded. The studies included: drug-induced metabolic syndrome, drug-induced severe extrapyramidal side effects, drug-induced liver injury, drug-induced neutropenia, and drug-induced malignant syndrome.

A total of 228 patients participated in this study. The inclusion criteria was: (1) patients were diagnosed Schizophrenia by at least two psychiatrists with strict training and rich clinical experience according to Diagnostic and Statistical Manual of Mental Disorders, Fourth Edition (DSM-IV). (2) All were treated with a single drug (One of olanzapine, risperidone, aripiprazole amisulapride and quetiapine during the research. (3) The patient (or guardian) has informed consent and is willing to participate in the study. The exclusion criteria were: (1) Taking other types of antipsychotics and antiepileptic drugs within 1 month. (2) Using stabilizers and antidepressants within 1 month. (3) Don't receive modified electric convulsive therapy (MECT) for 1 month. (4) Patients with genetic diseases. (5) patients with serious medical illness, impulsive intention or suicide attempt. (6) The patient is pregnant or lactation.

### SNPs selection

Single nucleotide polymorphism (SNP) selection criteria: (1) Derived from NPY gene; (2) Previous studies have confirmed that it is related to the occurrence of schizophrenia; (3) The SNP locus in Chinese han population of minimum allele frequency (minorallele frequency, MAF) > 0.1. SNPs are filtered in the following database: Database of Single Nuleotide Polymorphisms (dbSNP), International HapMap Project, SZDB database (http://www.szdb.org/). Three SNPs were screened out, namely rs16141, rs16145, and rs5573.

### DNA extraction and SNPs genotyping

A total of 5 ml whole blood from subjects collected in EDTA anticoagulant blood collection tubes and stored in −80°C refrigerator. The RelaxGene Blood DNA System was used to extract DNA and quantify the extracted DNA. SNP analysis was performed using the Kompetitive Allele-Specific PCR genotyping system (KASP).

### Drug intervention and clinical rating scale

Subjects were treated with a single antipsychotic drug for 8 weeks. The medications included risperidone (67 cases), olanzapine (98 cases), aripiprazole (41 cases), quetiapine (4 cases), and amisulapride (18 cases). Clinical symptoms and severity were assessed using the PANSS, which was, respectively assessed before treatment and on the weekends of the 2nd, 4th, and 8th weeks after treatment. Assessments were performed independently by 2 highly trained senior psychiatrists blinded to subject genotype. The change in the score of PANSS is used as the main evaluation index. We used percent change in PANSS to assess treatment response to antipsychotics. In this study, 8-week PANSS score was used as the PANSS endpoint score. Patients were sorted into the good responders if they had a reduction rate in PANSS score ≥50 % after 8 weeks, and were otherwise sorted into the poor responders.

PANSS percentage change = (PANSS baseline score - PANSS endpoint score)/(PANSS baseline score - 30) × 100

### Statistical analyses

Statistical analyses were carried out with the SPSS package (version 26.0). Age and PANSS baseline scores were compared between groups using independent samples *t*-test. The chi-square test was used to analyze the differences between the various qualitative data of the subjects, including gender, allele frequency and genotype frequency. The relevance between different SNPs genotypes and antipsychotic drug efficacy was calculated by Spearman correlation analysis. Bonferroni correction was applied in multiple testing. Genetic model analysis was performed using the SNPstats online tool. Haploview v4.0 was used to conduct linkage disequilibrium (LD) and haplotype analysis. The D' value is used to measure the degree of LD among the various loci of the NPY gene. The statistical significance level was *p* < 0.05 and all tests were two-tailed.

## Result

### Demographic and clinical characteristics

A total of 228 subjects were enrolled in this study which 192 patients were good responders and 36 were poor responders. There was no significant difference in gender (χ2 = 2.521, *p* = 0.112), age (*p* = 0.577) and PANSS baseline score (*p* = 0.051) between the two groups (Table 1).

**Table 1:** Descriptive statistics for patient-related variables with regard to response.

**Response**	**Good responders**	**Poor responders**	***P*-value**
Sex	Male (49%)	Male (64%)	0.112
	Female (51%)	Female (36%)	
Age	38.92 ± 11.64	37.73 ± 11.68	0.577
Baseline PANSS score	85.11 ± 6.73	87.23 ± 55.75	0.051

### Effects of NPY gene polymorphisms on antipsychotic treatment response

We calculated the PANSS total deduction rate for 2nd, 4th and 8th week, respectively. The analysis showed that three SNPs didn't had significant association with reduction in PANSS total score at different stages (all *p* > 0.05). The PANSS scale includes positive symptom scale, negative symptom scale and general psychopathological symptom scale. We calculated the positive score change, negative score change and general psychopathology score change at different stages. There was no significant relationship between the rs16141, rs16145, and rs5573 polymorphisms in the NPY gene and the changes of positive, negative and general psychopathology subscales score changes at each stage (all *p* > 0.05, Table 2).

**Table 2:** Association of NPY gene polymorphisms with antipsychotic treatment response.

**SNP**	**Total reduction rate**			**Positive score change**			**Negative score change**			**Psychopathology score change**		
	**8th week**	**4th week**	**2nd week**	**8th week**	**4th week**	**2nd week**	**8th week**	**4th week**	**2nd week**	**8th week**	**4th week**	**2nd week**
rs16141	0.674	0.935	0.986	0.099	0.154	0.530	0.485	0.758	0.509	0.711	0.565	0.699
rs16145	0.712	0.951	0.224	0.971	0.902	0.074	0.667	0.873	0.488	0.398	0.897	0.927
rs5573	0.894	0.954	0.318	0.61	0.76	0.17	0.516	0.956	0.397	0.678	0.658	0.785

The values in the table represent the p-value of the Spearman correlation between the reduction rate of PANSS and SNP polymorphism at different stages.

### Genotypic and allelic distributions of NPY genes in two groups

The genotypic and allelic distributions of rs16141 in NPY gene were significantly different between two groups (genotype: χ^2^ = 4.877, *p* = 0.043; allele: χ^2^ = 4.088, *p* = 0.027), while there was no significance after Bonferroni correction (*adjusted-p* > 0.05). No difference in the genotype distribution of rs5573 (χ^2^ = 5.736, *p* = 0.057), while the allele distribution of rs5573 was significantly different between groups (χ^2^ = 4.136, *p* = 0.042). When correcting for multiple testing, the difference of allelic distribution was no significance. There was no significant difference in the distribution of rs16145 alleles and genotypes between groups (genotype:χ^2^ = 1.099, *p* = 0.295; allele: χ^2^ = 2.197, *p* = 0.138). The comparison of allelic and genotypic distributions of three SNPs in NPY gene between good responders and poor responders are presented in,Tables 3, 4, respectively.

**Table 3:** Comparison of SNPs genotype distribution in two groups.

**SNP**	**Genotype**	**Good responder (%)**	**Poor responder (%)**	***χ^2^***	***P-value***	***Adjusted-p***
rs16141	TT	171 (89.1)	27 (75)	4.088	**0.043**	0.129
	GT	21 (10.9)	9 (25.0)			
rs5573	AA	93 (48.9)	14 (51.9)	5.736	0.057	0.171
	AG	77 (40.5)	13 (48.1)			
	GG	20 (10.5)	9 (0.0)			
rs16145	GG	91 (48.4)	14 (38.9)	1.099	0.295	0.885
	TT	97 (51.6)	22 (61.1)			

The adjusted p-value was corrected by Bonferroni. *p*-values in bold indicate significant for the association.

**Table 4:** Comparison of SNPs allele distribution in two groups.

**SNP**	**Allele**	**Good responder(%)**	**Poor responder(%)**	***χ^2^***	***P-value***	***Adjusted-p***
rs16141	T	363 (94.5)	63 (87.5)	4.877	**0.027**	0.081
	G	21 (5.5)	9 (12.5)			
rs5573	A	263 (69.2)	41 (56.9)	4.136	**0.042**	0.126
	G	117 (30.8)	31 (43.1)			
rs16145	G	182 (48.4)	28 (38.9)	2.197	0.138	0.414
	T	194 (51.6)	44 (61.1)			

The adjusted p-value was corrected by Bonferroni. *p*-values in bold indicate significant for the association.

### Linkage disequilibrium and haplotypes

**Figure 1:**
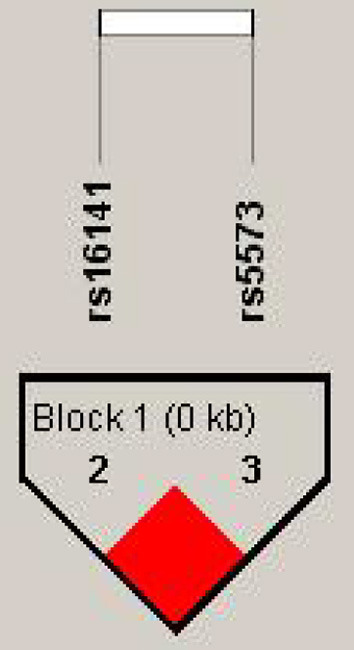
Linkage disequilibrium block structure across NPY gene. The figure show the output of Haploview (version 4.0) LD Plot where each square (with D^,^ values written within the box) represents a pair-wise LD relationship between the two SNPs. Red squares indicate statistically significant LD between the pair of SNPs as measured by the D^,^ statistic.

We detected linkage disequilibrium among rs16141, rs16145, and ra5573 in NPY gene and compared Haplotype frequency between good responder and poor responder. High LD was observed between SNPs rs16141-rs5573 and consisted a block (Figure 1). There was three different haplotypes in block1, namely TA, TG, and GG. The haplotype TA and haplotype GG were significantly associated with antipsychotic treatment response, while the significant association disappeared after 1,000 times permutations (rs16141-rs5573:TA,χ^2^ = 4.028, *p* = 0.0448, *adjusted-p* = 0.0860. rs16141-rs5573: χ^2^ = 4.877, *p* = 0.0272, *adjusted-p* = 0.0670, Table 5).

**Table 5:** Frequency distribution and association analysis of haplotypes of NPY gene with antipsychotics response.

**Haplotype**		**Haplotype frequency**	**Good responder (%)**	**Poor responder (%)**	***χ^2^***	***p*** **^a^**	***Adjusted-p*** **^b^**
rs16141	rs5573						
T	A	0.671	0.691	0.569	4.028	**0.0448**	0.0860
T	G	0.263	0.255	0.306	0.806	0.3693	0.6070
G	G	0.066	0.055	0.125	4.877	**0.0272**	0.0670

a Uncorrected p-value.

b Adjusted-p, number of permutations: 1,000. *p*-values in bold indicate significant for the association.

### Five genetic models analysis

After adjusting for sex and age, we further explored the relationship between three SNPs and antipsychotic drug response under five genetic models. Notably, rs5573 was associated with antipsychotic drug responses under five genetic models. In the recessive genetic models, taking AA+AG as a reference, the distribution of GG genotype is statistically significant difference between good responder and poor responder (G/G vs. A/A +A/G: *p* = 0.028, AIC = 197.2, BIC = 210.9). Genotype GG were more prevalent in poor responders than in good responders. After adjusting for age and sex, the associations remained significant in this model. However, after correction, this significance disappears after Bonferroni correction (*adjusted-p* = 0.0084) (Table 6).

**Table 6:** Logistic regression analysis of associations between the genotypes of NPY rs5573 with antipsychotic response.

**Model**	**Genotype**	**Poor responder (%)**	**Good responder (%)**	**OR (95% CI)**	***p*-value**	**AIC**	**BIC**
Codominant	A/A	14 (38.9%)	90 (48.4%)	1.00	0.088	199.2	216.2
	A/G	13 (36.1%)	77 (41.4%)	0.93 (0.41–2.11)			
	G/G	9 (25%)	19 (10.2%)	0.34 (0.13–0.90)			
Dominant	A/A	14 (38.9%)	90 (48.4%)	1.00	0.31	201.1	214.7
	A/G-G/G	22 (61.1%)	96 (51.6%)	0.69 (0.33–1.43)			
Recessive	A/A-A/G	27 (75%)	167 (89.8%)	1.00	**0.028**	197.2	210.9
	G/G	9 (25%)	19 (10.2%)	0.35 (0.14–0.86)			
Overdominant	A/A-G/G	23 (63.9%)	109 (58.6%)	1.00	0.55	201.7	215.3
	A/G	13 (36.1%)	77 (41.4%)	1.25 (0.59–2.64)			
Log-additive	—	—	—	0.62 (0.38–1.03)	0.066	198.7	212.3

AIC, Akaike Information Criterion; BIC, Bayesian information criterion. *p*-values in bold indicate significant for the association.

## Discussion

Based on the fact that NPY gene is an important schizophrenia susceptibility gene and may be associated with the efficacy of antipsychotic drugs, this study chose to explore the association between NPY gene polymorphisms and the efficacy of antipsychotic drugs. This study showed that the rs5573 locus may be related to antipsychotic treatment response in a recessive inheritance pattern while this difference disappeared after Bonferroni correction. We did not find an association between rs16141 and rs16145 and antipsychotic efficacy.

First, in this study, we didn't find that rs5573 was associated with antipsychotic treatment response. Few studies have reported the relationship between rs5573 and schizophrenia and drug efficacy. Rs5573 is located at the exon site of NPY gene. Zhou et al. (20)conducted the expression of NPY gene in lymphoblastic cells and found that the A allele of rs5573 involved in the formation of haplotype was related to the down-regulation of NPY gene expression. Zhang et al. (21) found that the G allele of rs5573 was included in five SNP haplotypes associated with elevated plasma NPY. These studies suggest that rs5573 may affect plasma NPY expression. Changing the expression level of NPY in the central nervous system may provide new ideas for the treatment of mental diseases in the future (22).

In the schizophrenia study, the messenger RNA and peripheral protein of the NPY gene were different between schizophrenics and healthy subjects. In 2009, Morris HM et al. (23) found that the mrna content of NPY decreased significantly in the surface white matter of schizophrenia group. Many studies have shown that plasma NPY levels increase after antipsychotic treatment in patients with schizophrenia. For example, AK et al. (24) found that the plasma NPY content of schizophrenia patients was lower than that of the control group. After 4 weeks of olanzapine treatment, the plasma NPY content of schizophrenia patients was higher than that before treatment. In an animal study (25), olanzapine and clozapine administration reduced NPY mRNA levels in the nucleus accumbens, striatum, and anterior cingulate cortex in rats. Haloperidol decreased NPY mRNA expression in amygdala and hippocampus. Kirk et al. (26) found that clozapine (but not haloperidol) increased NPY immune reactivity in the arcuate nucleus of the hypothalamus in rats. However, no studies have shown that changes in NPY levels are associated with antipsychotic efficacy. In this study, we found that there was a difference in rs5573 polymorphism between the two groups in recessive inheritance mode, but the difference disappeared after correction, further sample size expansion is necessary in the future to verify the accuracy of the results. Our findings have implications for the development of antipsychotic drugs and individualized treatment strategies.

This study found no significant association between the NPY gene rs16141 and rs16145 polymorphisms and the efficacy of antipsychotic drugs (PANSS score reduction rate). This may be due to the fact that these two sites are not related to the efficacy of antipsychotic drugs, but it may also be due to differences in the mechanism of action of olanzapine, risperidone, aripiprazole, and quetiapine, so these cannot be completely ruled out possibility of genetic loci associated with drug efficacy. At the same time, a review of previous literature found that there were few studies have explored the relationship between rs16141 and rs16145 and the efficacy of antipsychotic drugs. More studies on rs16141 and rs16145 and antipsychotic drug induced disorder of substance metabolism. Diaz et al. (27) conducted correlation analysis of acetyl-CoA carboxylase α, acetyl-CoA carboxylase β, NPY gene and blood lipid level in patients taking antipsychotic drugs in 2009. The SNP of NPY gene (rs16145, rs16478, rs16141) was not associated with serum cholesterol levels in patients taking olanzapine, quetiapine, or chlorpromazine, or in patients taking other antipsychotic drugs.

This study reports on the association of NPY with antipsychotic drug response, and our findings may provide new markers of drug response. The potential of NPY as a new drug target was studied. In recent years, the importance of neuropeptides in schizophrenia has been increasingly recognized due to their ability to modulate signal transduction of the monoaminergic neurotransmitter dopamine (28). In an animal study using disrupted-in-schizophrenia 1 (DISC1) knockout (KO) mice as an animal model of schizophrenia, we found a reduction in the number and fiber length of NPY-immunoreactive (NPY-IR) neurons in the prefrontal cortex of DISC1 KO mice, suggesting that DISC1 dysfunction may be involved in the pathogenesis of schizophrenia through impairment of the NPY neural network (29). Thiriet et al. (30) found that NPY promotes chemotaxis and neurogenesis of rat Subventricular zone (SVZ) cells through the Y1 receptor-mediated ERK1/2 MAP kinase pathway. Experiments on rodent brain cells have shown that NPY protects against the death of dentate gyrus cells and the decline in neurogenesis caused by methamphetamine abuse. NPY enhances neuronal proliferation and differentiation by binding Y1 receptor to activate MAPK pathway (31). The regulatory and protective effects of NPY on stress may play a role in psychopathology. Studies have shown that NPY neurons in hypothalamus, amygdala and hippocampus are sensitive to stress, and NPY regulates the stress response system by limiting the activity of corticotrophin releasing hormone (CRH) and norepinephrine (NE) (32). NPY also plays an important role in cognitive behavior, possibly by protecting cortical neurons from toxic effects of glutamate (33) and amyloid peptide (34). These studies support the role of NPY in the pathogenesis of schizophrenia and thus identify NPY as a potential drug target for the prevention and treatment of schizophrenia. However, an animal experiment (35) showed that no reduction in anxiety-related behavior and fear-learning behavior was observed in mice chronically overexpressed in NPY, possibly due to reduced NPY receptor reactivity caused by overexpression of NPY. Although this study is only about anxiety and stress response, it also has important reference significance for the study of schizophrenia and NPY.

There are some limitations to our study. First, due to the small sample size, it was not able to provide sufficient population genetic information, leading to the limitations of the results; Second, this study did not take into account some potential non-genetic factors, such as drug type, disease duration and drug dose, which affected the accuracy of the results to a certain extent. Third, we only tested it in the Chinese population, and it needs to be tested in other ethnic groups. In general, the association between NPY gene polymorphism and antipsychotic drugs is still worth further investigation. In future exploration, the selection range of SNP loci of NPY gene can be expanded, other races can be added, and drug types can be subdivided to obtain more convincing results.

## Conclusion

The aim of this study was to determine whether the NPY gene in schizophrenia affects antipsychotic treatment outcomes in Chinese Han population. In our study, none of the three SNPs (rs16141, rs16145, and rs5573) remained significant after Bonferroni correction. Future studies should be conducted in larger samples and diverse populations to better guide personalized medicine.

## Data availability statement

The datasets presented in this article are not readily available because of ethical and legal concerns. Requests to access the datasets should be directed to contact the corresponding author.

## Ethics statement

The studies involving human participants were reviewed and approved by Ethics Committee of First Hospital of Shanxi Medical University. The patients/participants provided their written informed consent to participate in this study.

## Author contributions

QZ: planned the study, performed the analyses, and wrote the manuscript. YW: data collection and genetic screening. YG, XD, and XW: conceptualization, writing-review and editing, and supervision. SL: conceptualization, funding acquisition, and writing-review and editing. YX: conceptualization and funding acquisition. KW, XZ, YW, CZ, LL, XL, and XG: data collection. All authors approved of the submitted version.

## Funding

This work was supported by the National Natural Science Foundation of China (81971601 and 81701326); the National Key Research and Development Program of China (2016YFC1307004); the Multidisciplinary Team for Cognitive Impairment of Shanxi Science and Technology Innovation Training Team, China (201705D131027); Shanxi Provincial Science and technology achievements transformation and guidance project (201904D131020); Shanxi Provincial Department of Education University Science and technology innovation plan project (202010203); 2021 Four Batch Medical Science and Technology Innovation Plan Medical Science and Technology Young Talents (2021RC24); Shanxi Provincial Health Commission Scientific Research Program Talents Special Project, Imaging Genetics of Magnetic Convulsive Therapy in Refractory Schizophrenia (2020081); Shanxi Scholarship Council of China; the National Natural Science Foundation of China (82271546).

## Conflict of interest

Author LL was employed by Yangquan Coal Industry Group Co., Ltd. The remaining authors declare that the research was conducted in the absence of any commercial or financial relationships that could be construed as a potential conflict of interest.

## Publisher's note

All claims expressed in this article are solely those of the authors and do not necessarily represent those of their affiliated organizations, or those of the publisher, the editors and the reviewers. Any product that may be evaluated in this article, or claim that may be made by its manufacturer, is not guaranteed or endorsed by the publisher.
